# Prevalence of latent tuberculosis infection and associated risk factors among 3,374 healthcare students in Italy

**DOI:** 10.1186/s12995-014-0034-5

**Published:** 2014-10-02

**Authors:** Monica Lamberti, Mariarosaria Muoio, Maria Grazia Lourdes Monaco, Rossella Uccello, Nicola Sannolo, Gennaro Mazzarella, Elpidio Maria Garzillo, Anonio Arnese, Giuseppe La Cerra, Nicola Coppola

**Affiliations:** Department of Experimental Medicine, Section of Hygiene, Occupational Medicine and Forensic Medicine, Second University of Naples, Naples, Italy; Department of Cardio-Thoracic and Respiratory Science, Second University of Naples, Naples, Italy; Department of Mental Health and Public Medicine, Second University of Naples, Naples, Italy

**Keywords:** Medical students, Tuberculosis, Tuberculin skin testing, Quantiferon test, Health surveillance, Occupational exposure

## Abstract

**Introduction:**

The risk of tuberculosis (TB) in healthcare personnel (HCP) is related to its incidence in the general population. Healthcare students involved in clinical training could be exposed to occupational risks similar to those that HCP face. The prevalence of latent tuberculosis infection (LTBI) among undergraduate healthcare students with different working seniority in Italy was analysed.

**Methods:**

A cross-sectional study under a screening programme for LTBI among undergraduate and postgraduate students attending Medical School at the Second University of Naples was conducted between January 2012 and December 2013 with clinical evaluations, tuberculin skin testing (TST) and, in positive TST students, Interferon-γ release assays (IGRA). Putative risk factors for LTBI were assessed by means of a standardised questionnaire.

**Results:**

3,374 students attending the Medical School of the Second University of Naples were submitted to a screening programme for TBC. 3,331 performed TST as a first-level test and 43 performed a Quantiferon test (QFT). 128 students were TST-positive and continued the diagnostic work with QFT, which was positive in 34 students. Of the 43 subjects who took the QFT as a first-level test only 1 was positive. In 35 students positive to the QFT test we formulated the diagnosis of LTBI by clinical and radiographic results. A correlation was found between age, non-Italian born persons, studying age, post-medical school status and LTBI.

**Conclusions:**

The prevalence of LTBI among healthcare students in our study was very low. In countries with a low incidence of TB, the screening programmes of healthcare students can be useful for early identification and treatment of sporadic cases of LTBI.

**Electronic supplementary material:**

The online version of this article (doi:10.1186/s12995-014-0034-5) contains supplementary material, which is available to authorized users.

## Introduction

According to the World Health Organization (WHO), there were an estimated 8.6 million cases of tuberculosis (TB) (range 8.3–9.0 million) globally in 2012, equivalent to 122 cases per 100,000 people. One third of the world’s population is estimated to be latently infected with *Mycobacterium tuberculosis*. People with latent TBC infection (LTBI) do not show symptoms of TB and are not infectious, but they are at risk of developing active disease and becoming infectious in about 10% of cases in the course of their lives. In around half of these cases, active TB develops within the first two years after infection. [1]. Malnutrition, homelessness and alcoholism, along with HIV infection and other diseases that weaken the immune system, or immunosuppressive therapies (in which TNF-alpha blockers are especially significant) are typical risk factors for activation of LTBI [2].

TB is a current risk even in low-income countries due to abandonment of vaccination campaigns, immigration flows, wide diffusion of primary or secondary immunosuppression and poor efficacy of vaccines currently in use [3,4]. All this makes tuberculosis a major health problem for public health.

The overall incidence of TB in Italy in recent years was 7.85 per 100,000 with approximately 4,400 new TB cases annually. In the past decade there has been a progressive decrease in its incidence in people aged over 60 and a slight and gradual increase in age groups from 15 to 64. [5] In healthcare personnel (HCP), the risk of TB infection is increased by exposure to patients with infectious disease, insufficient use of protective equipment such as respirators, and working conditions, particularly in inadequately ventilated areas and when conducting techniques that involve exposure to contaminated aerosols [6,7].

Healthcare students involved in clinical training could be exposed to occupational risks similar to those of HCP. In fact, the annual risk of TB infection (ARTI) among medical and nursing trainees was found to be 5% in countries with a high incidence of TB, 3 times higher than the 1.5% ARTI estimate for the general public. [8] LTBI screening of both healthcare workers and undergraduate students is recommended in low-incidence countries regardless whether or not there is knowledge of transmission, prevention, and diagnosis [9] in order to obtain an early diagnosis of cases and prevent progression to active disease. The main purpose of this study was to evaluate the prevalence of LTBI among undergraduate healthcare students, in a context of low endemicity such as Italy’s, and it found possible associations between the outcome of the screening tests and epidemiological variables.

## Material and method

From January 2012 a screening programme for TB was approved and carried out at the Second University of Naples, which can be classified as a “low-risk” institution according to the CDC guidelines [10]. According to this programme all students in their first, third and sixth year of medical school, students at health profession school in their first and third year and students of specialised schools in their first and third year are actively required to undergo TB screening. The students of the health profession school are nursing students, paediatric nursing students, student radiographers and midwifery students; the students of specialised schools are post-medical school students. All the individuals evaluated in this programme between January 2012 and December 2013 were enrolled in this cross-sectional study. For each individual, information on the following variables was collected using a standardised questionnaire: age, gender, time of study in healthcare setting (study age), previous exposure to TB, family history of TB, BCG vaccination, place of birth, prior TST, workplace and chest radiographic findings. BCG vaccination was verified by scars or vaccination records. Tuberculin Skin Test (TST) was performed by trained personnel following standard procedures. In brief, 0.1 mL (2 TU) of purified protein derivative (PPD, RT23; Statens Serum Institute, Copenhagen, Denmark) was injected. The TST was administered to the volar side of the forearm of the participants and read 72 to 96 hours after application. A positive TST was defined as an induration measuring ≥ 10 mm in healthy subjects [11]. The transverse diameter of the induration was measured by specialised personnel. All the TST-positive cases were also tested with an Interferon-Gamma Release Assay (IGRA; QuantiFERON® TB-Gold Cellestis, Carnegie, Australia) to confirm the diagnosis of LTBI because of its major specificity compared with conventional TST [12]. The subjects with positivity for TST and Quantiferon (QFT) test, were in the absence of clinical and radiographic signs of active tuberculosis considered to have an LTBI. The blood samples were treated as recommended by the manufacturer. Briefly, 1 ml of whole blood was sampled in each of the three QFT tubes containing either TB-specific antigen (ESAT-6, CFP-10 and TB7.7), no antigen (negative control) or mitogen antigen (positive control), tubes were incubated at 37°C overnight before centrifugation, and IFN-γ concentrations (IU/ml) were measured by ELISA following the manufacturer’s protocol. An IFN-γ ≥ 0.35 IU/ml (TB antigens minus negative control) was considered a positive test [13]. All the IGRA positive cases were carefully examined by an infectious diseases specialist and underwent chest radiography.

Individuals with a history of allergy, secondary immunodeficiency or pregnancy were offered the opportunity to take the QFT as a first-level examination. In fact, although in literature there is no evidence that adverse reactions to the Mantoux test can influence the course of pregnancy [14], in order to enhance the protection of pregnant students,QFT was used as examination of the first level to reduce the risk of allergic type reactions to TST and to avoid the pregnant woman having to undergo two diagnostic tests in case of TST positivity.

Furthermore, IGRAs may be more sensitive than TST in immunosuppressed individuals and those less affected by advanced immunosuppression [15]. Statistical analysis of data was performed using SPSS v.17.0 software. Continuous variables were summarised as mean and standard deviation and categorical variables as absolute and relative frequencies. Differences in the mean were evaluated by an unpaired Student t-test, and the Chi-squared test was applied to categorical variables. A p value of <0.05 was considered to be statistically significant.

All activities were performed in compliance with the Helsinki Declaration and current healthcare standards according to the recommendations of the Italian Ministry of Health [16]. All students included in the survey were informed by a physician about the rationale and aims of the survey and written informed consent was obtained. According to Italian legislation on the guidelines for observational studies, ethical approval for conducting this survey was unnecessary and on this basis cross-sectional studies did not require formal approval by local Institutional Review Boards [17]. Personal information on the subjects included in the study was protected according to Italian law [18].

## Results

All 3,374 subjects, students attending the medical school, those attending the health profession school and specialised doctors at the Second University of Naples evaluated in the TB screening programme from January 1, 2012 to December 31, 2013 were included in the study. The main demographic and epidemiological characteristics of all subjects enrolled are listed in Additional file 1: Table S1.

All subjects reported that they had no previous contact with a confirmed TB disease case. A history of a previous TST negative test and subsequently BCG vaccination was identified in 125 older subjects (mean age 29.7 (+3.7) years) included in the study, 82 females and 43 males. They were vaccinated as required by the previous italian DPR. n.465/2001 , then abrogated.

**Figure 1:**
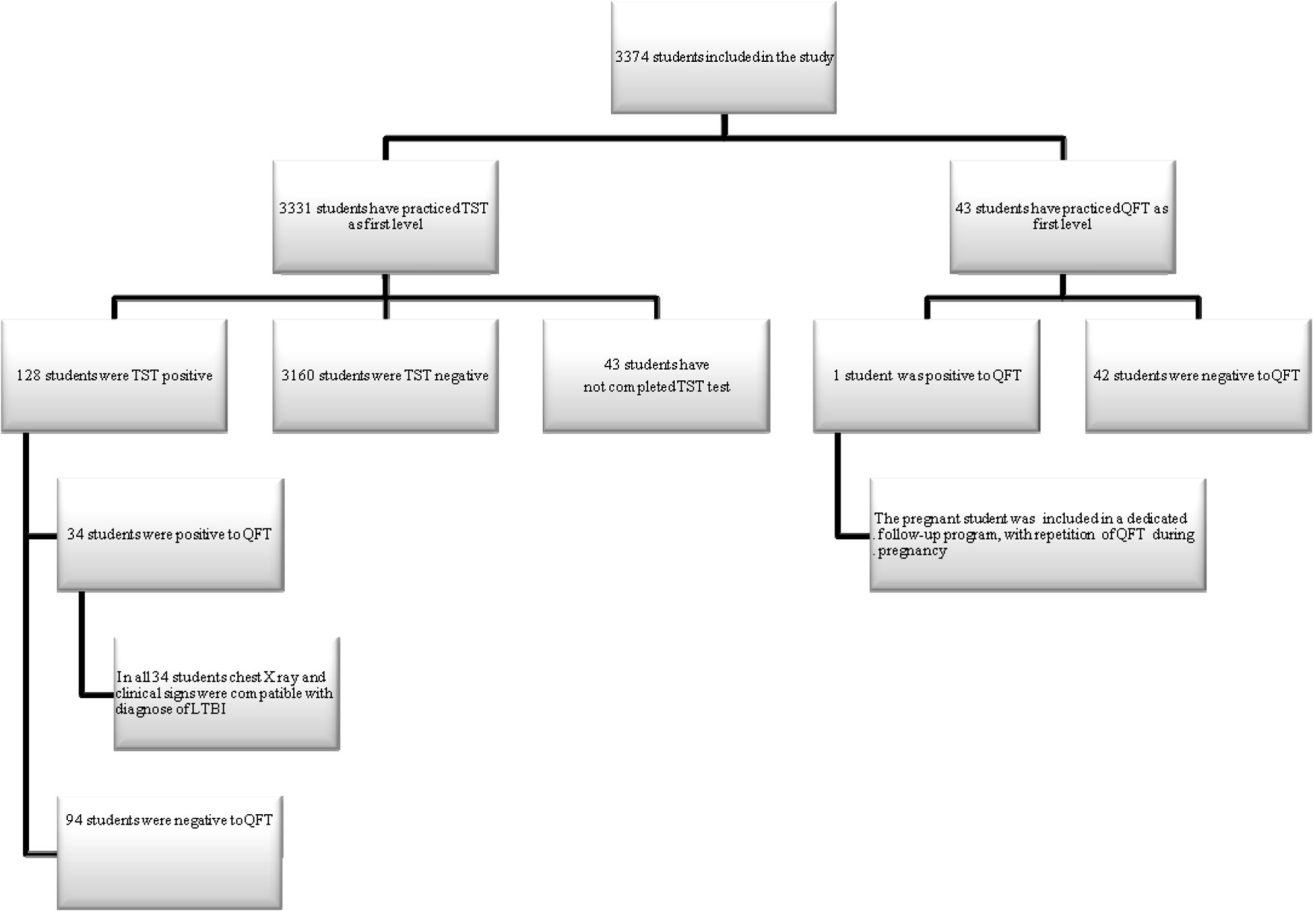
**Study flow chart.** **TST** Tuberculin Skin Test; **QFT** QuantiFERON® TB-Gold Test; **LTBI** latent tuberculosis infection.

Of the 3,374 subjects enrolled, 3,331 underwent TST as a first-level examination and 43 QFT (Figure 1). Of these 43, 22 had a positive history of drug allergy, 17 were pregnant and 4 were treated with immunosuppressive drugs. Of the 3,331 subjects who performed TST as a first test, 128 (3.8%) were positive, 3,160 (94.9%) negative and 43 (1.3%) were absent for reading in the window period from 72 to 96 hours after intradermal injection (20 women, 23 men, all Italian students). In these subjects we required to repeat the TST test after a month: it happened in 36 with negativity for TST, while the remaining 7 renounced to attend university and therefore have not performed a new test.

**Figure 2:**
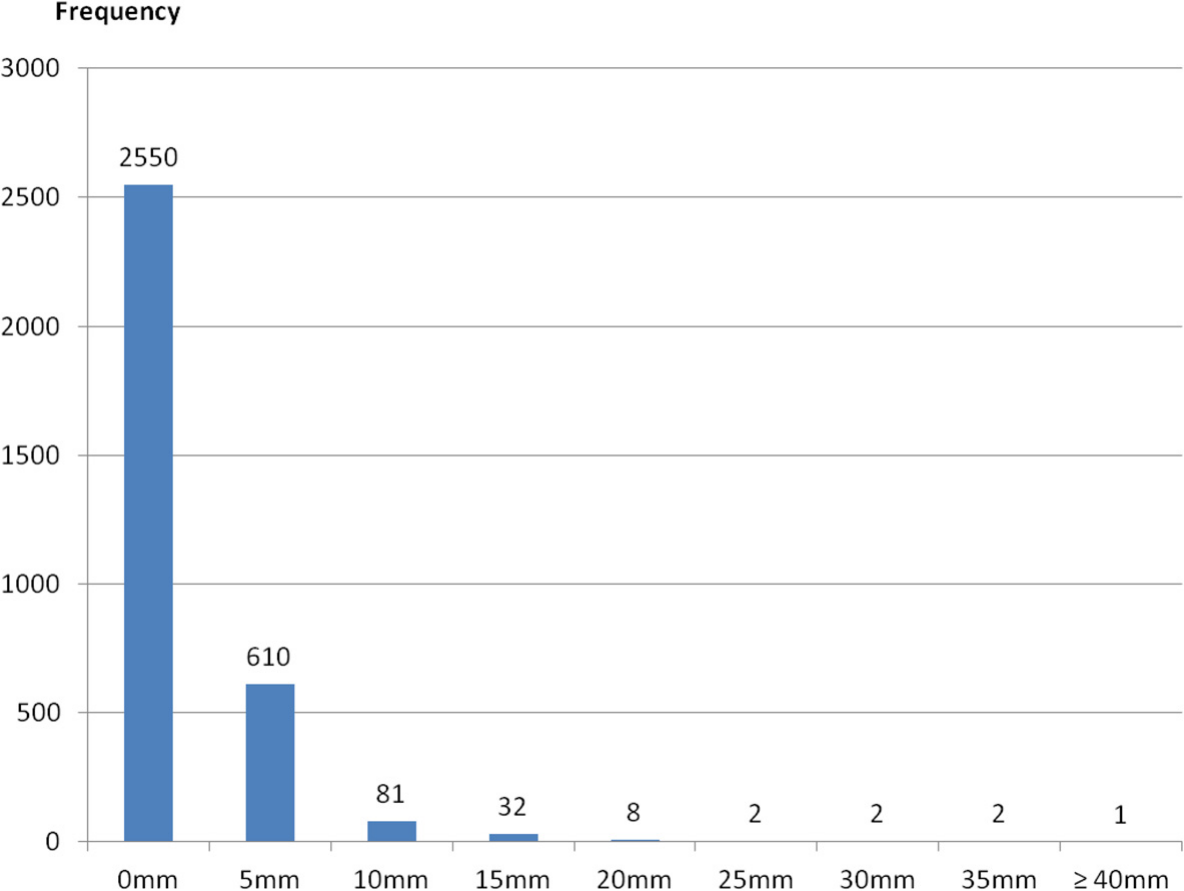
**Distribution of tuberculin skin test reactions (induration in mm).**

Regarding the safety profile of the tuberculin test, none of the individuals who took the test showed loco-regional or systemic allergic adverse reactions. Induration by TST ranged between 0 e 40 mm (Figure 2). All individuals with positive TST continued the diagnostic work-up by taking a QFT with the following results: 34 (26.5%) were positive and 94 (73.5%) negative. No significant correlation was observed between the size of TST induration and QFT positivity. Of the 94 subjects positive for TST but negative for QFT, 22 (23.4% of cases) had a history of BCG vaccination.

Of the 43 individuals who took QFT as first test, only one was positive. She was a pregnant medical specialist. She did not continue the diagnostic work-up with the diagnostic radiological techniques in accordance with the respiratory specialist and was included in a clinic dedicated follow-up programme. The 34 individuals tested positive for TST and QFT performed pulmonary consultation and chest X-ray. Clinical and radiographic signs of active tuberculosis were ruled out for all. Thus LTBI was identified in 35 of the 3,331 individuals tested (1.05%). Everyone was given an indication to undergo chemoprophylaxis. Only 8 students adhered to the drug treatment, and follow-up. Others 27 students refused a isoniazid preventive therapy (IPT).

Additional file 2: Table S2 shows the demographic characteristics of the 35 students with LTBI and the 3,253 without. Students with LTBI were older (31 + 6.3 years vs 25.5 + 5, p < 0.000), more frequently non-Italian born (8.6% vs. 0.5%, p < 0.000) and had a higher studying age 3.4 + 2.5 years vs 2.05 + 2.1, p < 0.000). A history of BCG vaccination was present (3.8% of cases) only in the group without LTBI. Instead, the individuals with LTBI were more frequently composed of students at postgraduate medical schools (42.9% vs. 25.6%, p < 0.05).

## Discussion

There is growing awareness of the problem of nosocomial TB and the need to protect healthcare workers in the era of multidrug-resistance (MDR) [19]. However, studies in industrialised countries have shown annual TST conversion rates ranging from 0.1% to 2% among unexposed personnel and from 0.5% to 14% among highly exposed HCP [20]. Programs for the screening and treatment of LTBI cases within HCP, combined with other interventions aimed at reducing the risk of nosocomial transmission, represent fundamental tools of TB control programmes and are strongly recommended in many countries with low TB incidence, including Italy [9,21].

The likelihood of developing active TB during the first two years following a positive IGRA is significantly lower for healthcare workers than for close contacts in the general population. This can probably be explained by the fact that healthcare workers have a low incidence of risk factors for developing active TB such as for example alcoholism, homelessness and that a positive IGRA in healthcare workers is often caused by an remote LTBI with a low risk of progression [3].

Students and assistants in training are considered potentially exposed to hospital infections as HCP [22]. Studies performed in high-incidence countries have reported LTBI prevalence figures ranging from 9.2% to 72% among healthcare students [23–26]. Our findings showed a prevalence of TST positive cases among all subjects of only 3.9%. Moreover, the diagnosis was confirmed by IGRA testing in only 35 students. Thus the prevalence of LTBI in this cohort was 1.05%. These results tally with the data observed among healthcare students at the beginning of the training period in various occupations (nurses, medical students, physiotherapists) by other authors in Europe [27–29] and are justified by the low risk of contact with infected patients in hospitals for the low prevalence of disease in industrialised countries.

Therefore, based on our data and in view of the low incidence of positive TST students, a risk evaluation every two or three years in absence of a new exposure appears to be sufficient to ensure workers’ health. However, a specific study on the periodicity of risk evaluation seams to need to clarify this point.

A higher age and studying age, to be non-Italian born and a postgraduate student at medical school seem to be factors associated with LTBI.

The role of age in influencing the TST positivity risk is controversial. Some studies revealed a significant positive association between age and TST conversion, [30,31] whereas others did not confirm this association [32,33]. In this study, the association between LTBI and a higher age, studying age and being a postgraduate student at medical school seems to suggest a higher risk of TB exposure in students with a longer stay in healthcare settings.

The higher prevalence of LTBI in non-Italian students reflected the epidemiology of TB. In our sample, the percentage of latent forms among foreigners was 14% versus 1% in the Italian group. All overseas students with LTBI come from countries where TB is endemic (Brazil, Ecuador, Eritrea, Mexico, Moldova, Russia, Ukraine). As shown in literature [34,35], the prevalence and incidence of tuberculosis in the country of origin can affect the outcome of the screening test. In countries with high endemicity that are often those with higher immunization coverage, we expected a high rate of positive skin test results, so that for workers from these countries the performance of QFT as a first-level test may be taken into account.

Considering the 93 subjects with positivity for TST and negativity for QFT, 21 had a history of BCG vaccination, while in the others 72 a possible cause of the positive TST test could be contact with non-tuberculous mycobacteria [36].

The tuberculin skin test in our experience is safe and widely used (no case of loco-regional or systemic allergic reactions) and the use of IGRA in TST-positive students reduces the potential occurrence of “false positive” cases due to exposure to atypical mycobacteria or BCG vaccination. However, both the TST and IGRA tests have limitations in diagnosing LTBI. The main problems with TST depend on technical limitations, difficulty in interpreting the results and the existence of a significant number of false positives [37,38]. On the other hand, IGRA tests, despite being more specific and having at least identical sensitivity to TST [39–41], present difficulties in interpreting results near the cut-off between positive/negative and also have higher unit costs [42]. Therefore our choice of using IGRA test as second-level examination in positive TST students was aimed at optimising costs and increasing the level of diagnostic accuracy.

## Conclusion

In this study we evaluated the incidence of TB in classes of healthcare students and postgraduate students at medical school with different studying ages. We confirm a low prevalence of LTBI among undergraduate and postgraduate healthcare students in our region. So in countries with a low incidence of TBC such as Italy a LTBI two-step screening strategy for healthcare students before and during clinical training seems justified [43].

## Additional files

Additional file 1: Table S1. Demographic, epidemiological and clinical characteristics of all the healthcare students enrolled trained at the Second University of Naples in Italy. Additional file 2: Table S2. Demographic, epidemiological and clinical characteristics of healthcare students enrolled, with completely performed TST and/or QFT test, trained at the Second University of Naples in Italy.
