# Supplementary material for: Prevalence of latent tuberculosis infection and associated risk factors among 3,374 healthcare students in Italy
Source: J Occup Med Toxicol. 2014 Oct 2;9:34. doi: 10.1186/s12995-014-0034-5 (PMC4190494; doi:10.1186/s12995-014-0034-5)
Supplement: Additional file 1: Table S1. — Demographic, epidemiological and clinical characteristics of all the healthcare students enrolled trained at the Second University of Naples in Italy. [file 12995_2014_34_MOESM1_ESM.doc]

**Table S1** Demographic, epidemiological and clinical characteristics of all the healthcare students enrolled trained at the Second University of Naples in Italy

|  | TOTAL | MS | NS | MSD |
| --- | --- | --- | --- | --- |
| Students included in the study (%) | 3,374 (100) | 939 (27.8) | 1,577 (46.7) | 858 (25.4) |
| Age of students, mean ± SD | 25,76 ± 5,3 | 25,4 ± 3,6 | 23,6 ± 5,4 | 30 ± 4,1 |
| Females, n° (%) | 2,021 (59.9) | 498 (53) | 1,020 (64.7) | 503 (58.2) |
| Non-Italian students | 21 (0.6) | 2 (0.2) | 10 (0.6) | 9 (1) |
| Studying age, mean ± SD | 2,1 ± 2,2 | 2,2 ± 1 | 0,4 ± 0,8 | 5,2 ± 1,2 |
| With history* of BCG vaccination, n (%) | 125 (3.7%) | 37 (3.9%) | 9 (0.6%) | 79 (9.2%) |
| TST, n° (%) |  |  |  |  |
| Tested | 3,288 (97.5) | 912 (97.1) | 1,562 (99) | 814 (94.9) |
| • positive | 128 (3.9) | 24 (2.6) | 19 (1.2) | 85 (10.4) |
| • negative | 3,160 (96.1) | 888 (97.4) | 1,543 (98.8) | 729 (89.6) |
| TST and QFT positive | 35* (1.06) | 8 (0.88) | 12 (0.76) | 15* (1.84) |

MS: students attending medical school; NS: nursing students, paediatric nursing students, student radiographers and midwifery students; MSD:Medical specialising doctors; SD: standard deviation; * BCG vaccination verified by scars or vaccination records.

*: One student with QFT positive as first test.
