# Supplementary material for: Prevalence of latent tuberculosis infection and associated risk factors among 3,374 healthcare students in Italy
Source: J Occup Med Toxicol. 2014 Oct 2;9:34. doi: 10.1186/s12995-014-0034-5 (PMC4190494; doi:10.1186/s12995-014-0034-5)
Supplement: Additional file 2: Table S2. — Demographic, epidemiological and clinical characteristics of healthcare students enrolled, with completely performed TST and/or QFT test, trained at the Second University of Naples in Italy. [file 12995_2014_34_MOESM2_ESM.docx]

**Table S2** Demographic, epidemiological and clinical characteristics of healthcare students enrolled, with completely performed TST and/or QFT test, trained at the Second University of Naples in Italy

|  | Students with LTBI | Students without LTBI | p |
| --- | --- | --- | --- |
| N° | 35 | 3,296 |  |
| Age, mean ± SD | 31 ± 6.3 | 25.5 ± 5 | 0.000 |
| Females, n° (%) | 20 (57.1) | 1981 (60.1) | 0.99 |
| Non-Italian students, n° (%) | 3 (8.6) | 18 (0.5) | 0.000 |
| Studying age, mean ± SD | 3.4 ± 2.5 | 2.05 ± 2.1 | 0.000 |
| With history* of BCG vaccination n (%) | 0 | 125 (3.8) | 0.4 |
| Type of school |  |  |  |
| - MS | 8 (22.8) | 920 (28.3) | MS + NS |
| - NS | 12 (34.3) | 1,543 (47.4) | vs. MSD |
| - MSD | 15 (42.9) | 833 (25.6) | <0.05 |

MS: students attending the Medical School; NS: nursing students, paediatric nursing students, student radiographers and midwifery students; MSD:Medical specializing doctors; SD: standard deviation; * vaccination verified by scars or vaccination records.
